# Supplementary material for: Super-resolution microscopy based on the inherent fluctuations of dye molecules
Source: Biomed Opt Express. 2025 Feb 4;16(3):910–21. doi: 10.1364/BOE.533263 (PMC11919343; doi:10.1364/BOE.533263)
Supplement: Supplementary file 1 [file boe-16-3-910-s001.pdf]

Supplemental Document

Biomedical Optics EXPRESS

Super-resolution microscopy based on the inherent fluctuations of dye molecules: supplement

ALEXANDER KRUPINSKI-PTASZEK,[1,5] ADRIAN MAKOWSKI,[1,2] ALEKSANDRA MIELNICKA,[3] MONIKA PAWŁOWSKA,[1] RON TENNE,[4,6] AND RADEK LAPKIEWICZ[1,7,]

[1] *Faculty of Physics, University of Warsaw, Pasteura 5, 02-093 Warsaw, Poland*
[2] *Laboratoire Kastler Brossel, ENS-PSL Université, CNRS, Sorbonne Université, Collège de France, 24 rue Lhomond, Paris 75005, France*
[3] *The Nencki Institute of Experimental Biology, PAS, 02-093 Warsaw, Poland*
[4] *Department of Physics, University of Konstanz, Universitätsstraße 10, D-78457 Konstanz, Germany*
[5] *a.krupinski-ptaszek@uw.edu.pl*
[6] *ron.tenne@uni-konstanz.de*
[7] *radek.lapkiewicz@fuw.edu.pl*

This supplement published with Optica Publishing Group on 4 February 2025 by The Authors under the terms of the Creative Commons Attribution 4.0 License in the format provided by the authors and unedited. Further distribution of this work must maintain attribution to the author(s) and the published article's title, journal citation, and DOI.

Supplement DOI: https://doi.org/10.6084/m9.figshare.28052243

Parent Article DOI: https://doi.org/10.1364/BOE.533263

Super-Resolution Microscopy Based on the Inherent Fluctuations of Dye Molecules: supplemental document

1. DATA ANALYSIS

The time-tagged data of photon detections (see Methods section) is parsed into the lines and pixels of the scan based on the stage trigger marker time. Detection in each scan pixel are then binned according to their detection time to generate microsecond-scale intensity traces (number of detected photons per 10 microsecond time bin). This results in a (23, X, Y, T) dataset, where X and Y are the image dimensions in pixels, and T is the number of ten-microsecond time-bins per scan position, typically few thousands. There are 23 X by Y pixel image series of duration T, with 50 nm spatial resolution (step size) and 10 μs temporal resolution. A CLSM image (Fig. S1 a) is obtained by summing the data across the first and last dimension, i.e. by treating the entire array as a single detector and summing all detection during the acquisition duration. To obtain an ISM image (Fig. S1 b), the data is first summed across the fourth (time) dimension, and pixel reassignment is performed prior to summation. Note that the reassignment vectors were pre determined according to a calibration measurement of an isolated fluorescent nanobead, where the center of a 2D gaussian function is estimated for each of the 23 shifted CLSM images, resulting in 23 shift vectors s_i with respect to the central detector element. For SOFISM, the temporal correlation $G^{(2)}(\tau)$ [1] between each detector pairs is calculated for each of the (x, y) sample positions, which results in a (23, 23, X, Y, D) dataset. The first (second) dimension corresponds to the correlation values between the first (second) detector and all other detectors, the third (fourth) dimension corresponds to the first (second) dimension of the scan grid, and the last dimension D corresponds to the time-delay of the correlation function. The data is then summed across the fifth dimension in a range corresponding to delays of up to 50 microseconds, resulting in 23^2 images. Then, pixel reassignment is performed, where each of the 23^2 images is shifted by the corresponding $s_{i,j} = \frac{s_i+s_j}{2}$ vector, which is the average of ISM shift vectors s_i and s_j for SPAD array pixels i and j. The shifted images are then summed to obtain the super-resolved SOFISM image (Fig. S1 c). Volumetric imaging is achieved slice by slice.

In diffraction-limited imaging, the information present at high spatial frequencies is attenuated due to the decay of the Fourier transform of the point-spread function (PSF), the optical transfer function (OTF). SOFISM can be thought of as taking integer powers of the CLSM PSF, corresponding to convolutions of the CLSM OTF. Consecutive convolutions further decrease the value of the OTF at high spatial frequencies, limiting the transfer of high frequency contents through the system. To enhance the high spatial frequencies in the SOFISM images, Fourier reweighting is applied obtaining an FR SOFISM image (Fig. S1 d). Fourier reweighting is done as follows:

1. 2D Gaussian PSF of width σ is generated
2. Fourier transforms of the image and PSF ($\tilde{I}$ and OTF, respectively) are computed
3. A filter is constructed $F = \frac{1}{OTF+\epsilon\frac{k}{k_{max}}}$, with $F(k > k_{max}) = 0$, where k are the spatial frequencies
4. The FR SOFISM image is obtained as the absolute value of the inverse Fourier transform of $F \cdot \tilde{I}$

Careful choice of the parameters is necessary to avoid deconvolution artifacts, with the ϵ depending on the SNR of the image, and k_{max} on the expected resolution. For high SNR data, the ϵ should be smaller. In Fig. 1 and Fig. 3, Fourier reweighting was performed using $k_{max} = 10$, $\epsilon = 0.5$ and $\sigma = 0.21 \cdot k_{max}$, whereas in Fig. 4e and Fig. 4g $\epsilon = 2$. The parameter values used for FR differ between measurements presented in Fig. 1 and Fig. 3 and those used for Fig. 4. The latter was acquired with shorter dwell time, which in turn led to lower SNR. To properly

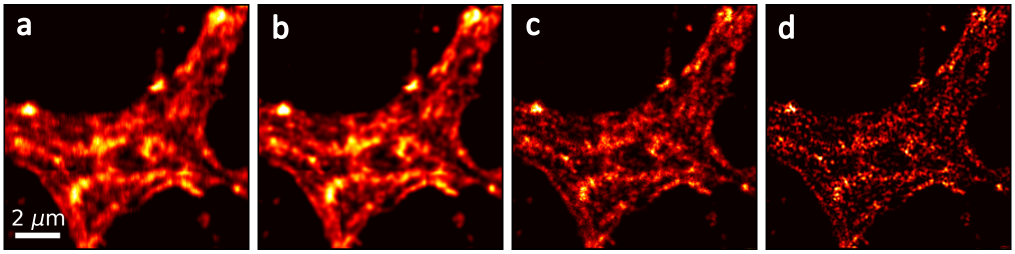

Fig. S1. Multiple image construction protocols from a single dataset. From left to right, consecutive columns present CLSM, ISM, SOFISM and FR SOFISM images for the imaging of F-actin in an astrocyte cell presented in Fig. 2 of the main text. Pixel size is 50 nm.

estimate the resolution of FR SOFISM in biological samples, We used the same FR parameter values when analyzing the QD data (Fig. S3) as for the high SNR neuron data (Fig. 1-3 in the main text). The QD dataset is characterized by high SNR and well-separated point-like emitters. Both of these factors reduce the likelihood of inducing deconvolution artifacts, and while tuning the FR parameters on the QD dataset would likely lead to higher resolution, it would not reflect a realistic experimental scenario of imaging a more complex, biological structure.

Bilinear interpolation followed by spline interpolation was used to obtain the line profiles presented in the main text. Bilinear interpolation is often used to approximate values of a function of two variables on a rectilinear grid (e.g. positions in an image), and it performs linear interpolation in first dimension, followed by the same operation in the second dimension. More precisely, it calculates the number of equally-spaced pixels of the same size as the original grid and calculates their coordinates. The values are then calculated as the average of the values at 4 discrete scan grid positions, weighted by the distance of the point to these positions. The bilinear interpolation results are always presented as points (squares), and only then spline interpolation is used to obtain a line profile, and the results are identical to the profiles obtained using ImageJ's Plot Profile tool with default settings.

2. CORRELATION CURVE

The contrast in SOFI originates from the temporal correlation of stochastic blinking of fluorophores [2]. In the case of dye molecules, the second-order intensity correlation decays with delay time in the timescale of the transition in and out of the triplet (dark) state. As described in the main text, we use the integral over the first $50\mu s$ (excluding 0 delay) as the image contrast. The average decay curve, shown in Fig. S2, is a robust diagnostic tool for new experimental conditions, such as staining protocols, measurement and data processing parameters, or hardware modifications. While the curve is not a mono-exponential one, we can observe a fast decay around $\tau = 0$ with a lifetime of $100\mu s$, followed by a slower decay on the millisecond scale. We recognize the former as the triplet-state lifetime of the Atto643 molecules.

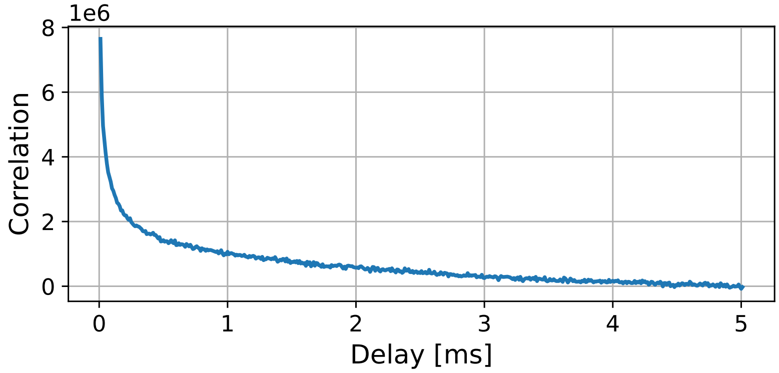

Fig. S2. Correlation for dataset presented in Fig. 1 in the main text. The plot represents the sum of time-dependent correlation summed over all pixels of image presented in Fig. 1 in the main text. The delay covers a range of 10 μs to 5 ms, in steps of 10 μs. The non-delayed correlation is omitted, and the first 5 delays (10 μs to 50 μs) at each image pixel are summed and used as the SOFI signal.

3. ESTIMATING THE IMAGING RESOLUTION

This section provides details on the determination of transverse resolution in the different imaging modalities shown within this work. For this purpose, we imaged 45 sparse scenes of quantum dots (see Methods section). These were then analyzed, using the above-mentioned protocols, to generate CLSM, ISM, SOFISM and FR SOFISM images for single isolated QDs (see examples in Fig. S3). Additionally, wide-field imaging of the same samples was performed to estimate the diffraction-limited resolution. Fitting the images to a 2D Gaussian function we determine the average FWHM of each method provided in the main text. We estimated resolution of WF, CLSM, ISM, SOFISM and FR SOFISM as 294 nm, 234 nm, 195 nm, 141 nm and 114 nm, respectively. Overall, FR SOFISM improves the lateral resolution beyond the diffraction limited wide-field imaging by a factor of 2.58, outperforming CLSM by a factor of 2.09.

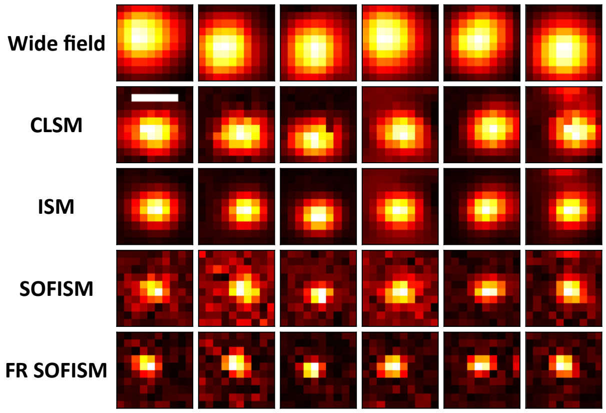

Fig. S3. QD data for transverse resolution estimate. Each column shows a small scene around an isolated QD ($0.5\mu m \times 0.5\mu m$). From top the bottom the rows present the result of wide-field, CLSM, ISM, SOFISM and FR SOFISM analysis for the same dataset. Scale bar: 300 nm.

Additionally, we performed decorrelation-based resolution estimation on data from a fixed-cell sample (Figure 2 in the manuscript). In the standard Fourier Ring Correlation (FRC) analysis, two independent images of an identical FOV are correlated, which often in scanning techniques are produced from the same dataset by dividing the acquisition time per pixel. However, we found that the standard 2D FRC analysis did not yield meaningful resolution estimates for intensity-based imaging modalities (CLSM and ISM) within the same dataset. To compare the

resolution of intensity-based and correlation-based methods using the same methodology, we have applied a 1D analysis by correlating pairs of consecutive lines to estimate the resolution along the scan direction ("Axis 2", Fig. S4b) and the orthogonal axis ("Axis 1", Fig. S4a). We have found that the analysis along Axis 1 results in high correlation values across the entire Fourier spectrum (Fig. S4a). At the same time, the correlation along Axis 2 (scan direction), while not decaying to 0 at high frequencies, contains a significantly lower background. The observed correlation, beyond the cut-off frequencies, reflects a similarity of noise between the two datasets, possibly stemming from mechanical stability and positioning accuracy in a raster scanning measurement, or photobleaching effects. Since the scan direction axis is more sensitive to such artifacts, below we use the orthogonal-to-scan axis (Axis 2) to estimate resolution. To address the issue of correlation background, we scale the correlation values to the range between 0 and 1 (See Fig. S4c).

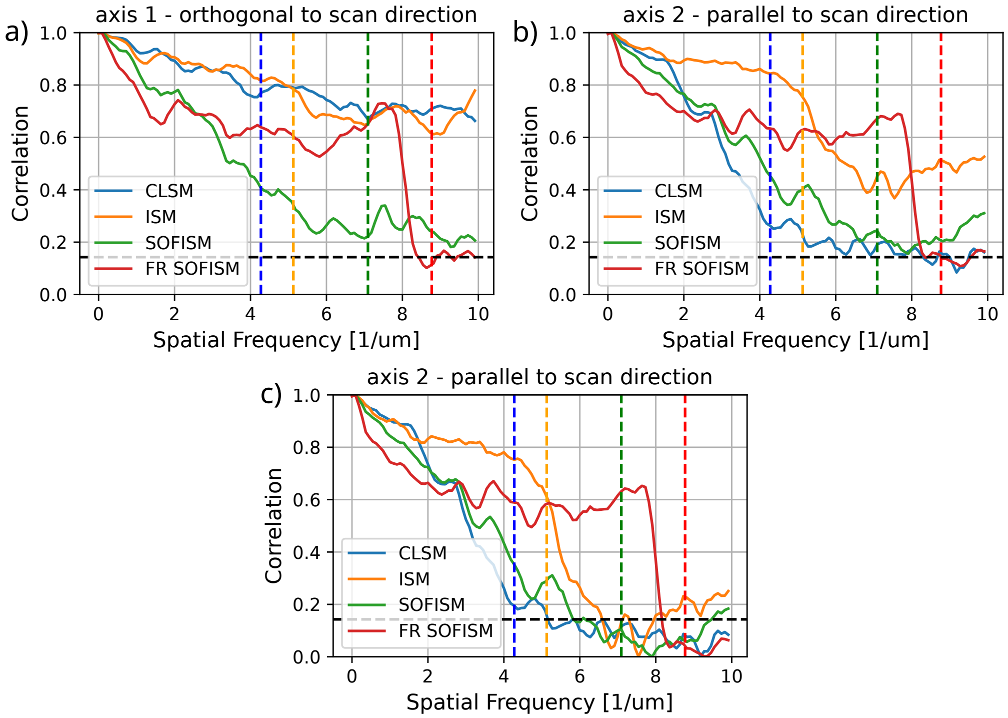

Fig. S4. Average correlation between consecutive lines.. (a) The axis orthogonal to the scan direction shows oscillatory components and significant background for intensity-based contrast of CLSM and ISM. (b) The axis parallel to the scan direction, while not free of background or oscillatory behaviour, shows a more plausible frequency response for CLSM, where it gradually decreases towards a physically attainable resolution. ISM frequency response shows an expected behaviour – increasing the magnitude of frequency components until cut-off frequency. (c) The values for axis 2 are scaled to range [0,1] to yield resolution estimates. The dashed vertical lines mark the resolution presented in the main text based on FWHM PSF measurements. The dashed horizontal lines mark the 1/7 threshold.

Adopting the typical 1/7 threshold, the values for CLSM, ISM, SOFISM and FR SOFISM are 234 nm, 159 nm, 177 nm, and 121 nm, respectively. In comparison, PSF fit estimates for the FWHM are 234 nm, 195 nm, 177 nm and 121 nm, in the same order. Using the correlation criterion, the overall resolution improvement estimate is 1.94, as compared to 2.05 derived with FWHM estimates.

4. AXIAL SECTIONING

Optical sectioning is one of the key features of confocal microscopy, as it enables volumetric imaging. As shown in Fig. 3 of the main text, SOFISM further improves the optical sectioning of CLSM. However, a fair comparison between the two methods requires the compensation of luminscence photo bleaching effects that deteriorate the CLSM and ISM images. The overall

signal per lateral slice recorded during the acquisition of 3D data exhibits a continuous decay with a time constant of roughly 24 minutes.

The signal recorded at the beginning of the measurement sequence appears brighter than during later stages of the experiment, as shown in the maximum-intensity projections (MIPs) of raw CLSM data presented in Fig. S5a, d. The top layers exhibit high brightness decaying towards the lower regions, which correspond to later measurement times. This artifact can be mitigated by fitting the intensity values integrated over each 2 μm by 2 μm lateral slice of the measurement to an exponential function, as shown in Fig. S5g (red line). This data is used to estimate the overall bleaching trend as a sum of two exponential decays (black dashed line). Consequently, a correction is applied by multiplying each of the intensity slices by the inverse function of the exponential estimated value, which results in the corrected axial intensity traces shown in Fig. 3f of the main text. The position of the maximum of this corrected trace coincides with the position of the peak visible in the raw data trace, which is mostly obscured by the exponential decay. MIPs of bleaching-corrected data for the same regions shown in Fig. S5a, d are presented in Fig. S5b, e, respectively. Here, the main feature can be clearly localized slightly below the center of each image. Fig. S5c, f present MIPs of the SOFISM signal for the same scenes without any correction applied. These demonstrate a finer resolved features in the same regions. The integrated signals for each lateral slice of $1\mu m \times 1\mu m$ containing the main feature in the images are shown in Fig. S5 h, highlighting the increased optical sectioning of SOFISM (red) compared to bleaching-corrected CLSM (black). It is worth noting that since the SOFISM contrast is based on correlation of the intensity fluctuations, it is less prone to photobleaching artifacts, and requires no bleaching-correction procedures to unveil the features obscured by the prominent, out-of-focus signal present in CLSM. Lastly, Fig. S5i presents the z-sectioning analysis for the XZ scans of an isolated QD presented in Fig. S5a of the main text. As QDs are significanly more photostable than organic dyes, no bleaching correction was necessary in this case.

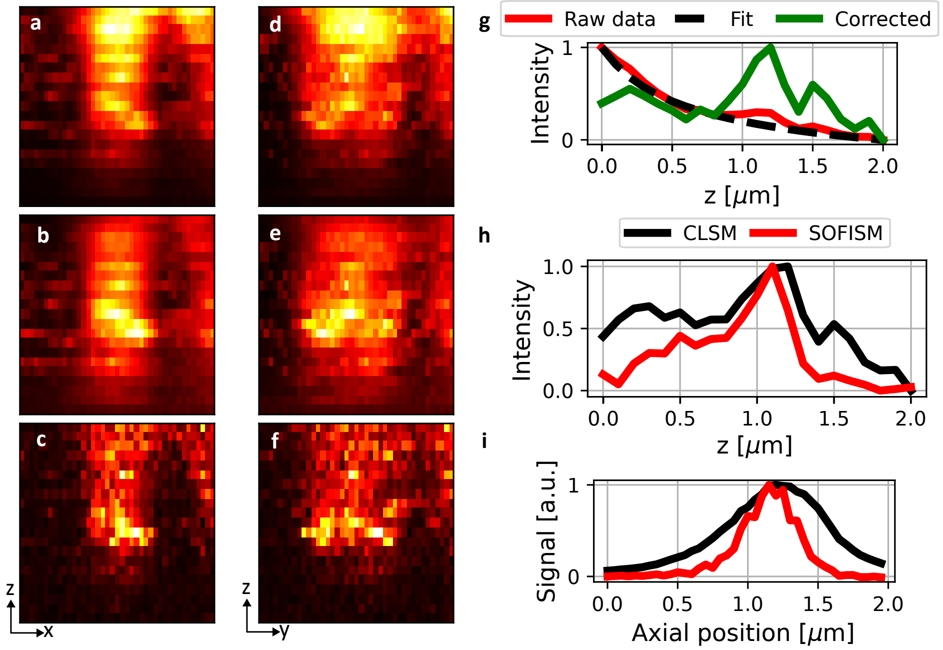

Fig. S5. Z-sectioning in SOFISM. (a, d) Maximum intensity projections (MIPs) of the raw CLSM signal. (b, e) MIPs of the bleaching-corrected CLSM signal. (c, f) MIPs of the SOFISM signal. g) Normalized intensity recorded at each lateral plane (red), double-exponential fit (black, dashed) and the corrected intensity (green). The correction unveils the position of the main feature, obscured by the exponential decay caused by photobleaching. h) Corrected intensity (CLSM, black) and correlation (SOFISM, red) values integrated over the lateral sections containing the main feature of the dataset. The SOFISM signal exhibits a sharper peak due to superior axial sectioning. i) Sectioning analysis for the quantum dot data presented in Fig. 3 of the main text.

5. CO-LOCALIZATION IN SOFISM

One of the main advantages of the multi-color SOFISM approach, presented in the main text, is the simultaneous acquisition of data for each position in the super-resolved image. In life-science imaging, often it is the co-localization of molecules and structures that is of interest to understand function. Super-resolved co-localization is a clear advantage of our approach. Fig. S6 presents a practical example of pulsed-interleaved excitation (PIE) for multicolor SOFISM in a primary rat neuronal cell culture. It shows a super-resolved co-localization of MAP2 (Alexa Fluor 488) and Homer1 (constitutes a major part of the postsynaptic density, stained with Atto 647N).

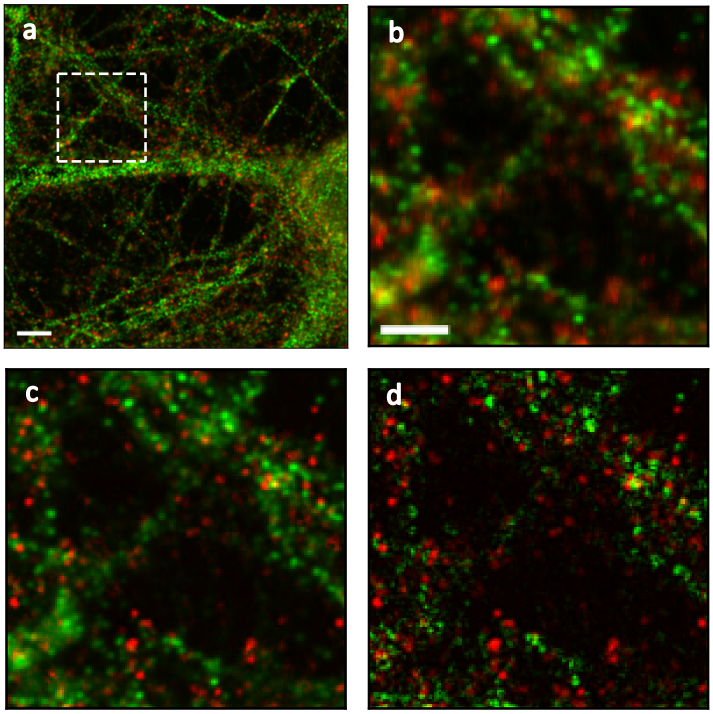

Fig. S6. Super-resolved co-localization of MAP2 and Homer in SOFISM. (a) CLSM. (b) CLSM of region marked with the dashed square. (c) ISM and FR-SOFISM performed in the green (MAP2) and red (Homer) channels, respectively (d) FR-SOFISM in both channels. Pixel size is 100 nm in (a), and 50 nm in (b)-(d), scale bars are 4 μm in (a) and 2 μm in (b)-(d).

6. THE EFFECT OF LABELING DENSITY ON SOFISM

The density of fluorescent labels in a biological sample is a universal problem in super-resolution microscopy (SRM). Structures that appear continuous in diffraction-limited images may become discontinuous as the resolution increases simply due to the sparsity of labels. This challenge, present in early demonstrations of most SRM techniques, becomes less restrictive as researchers develop and master staining procedures for various specimens and labels, throughout the years.

Fig. S1 showcases the gradual increase in perceived granularity as the resolution increases from CLSM to FR-SOFISM. It is important to note that granularity is not introduced by any specific step of the analysis to exclude, for example, the absence of SOFI contrast in dense areas. In comparison, in the datasets in Fig. S7, the structures in these examples remain relatively continuous even in the SOFISM image. While these do not represent a systematic solution to dense labeling and meeting the Nyquist criterion, they demonstrate that the issue is unlikely inherent to the SOFISM contrast but rather a lack of experience on our part in labeling protocols.

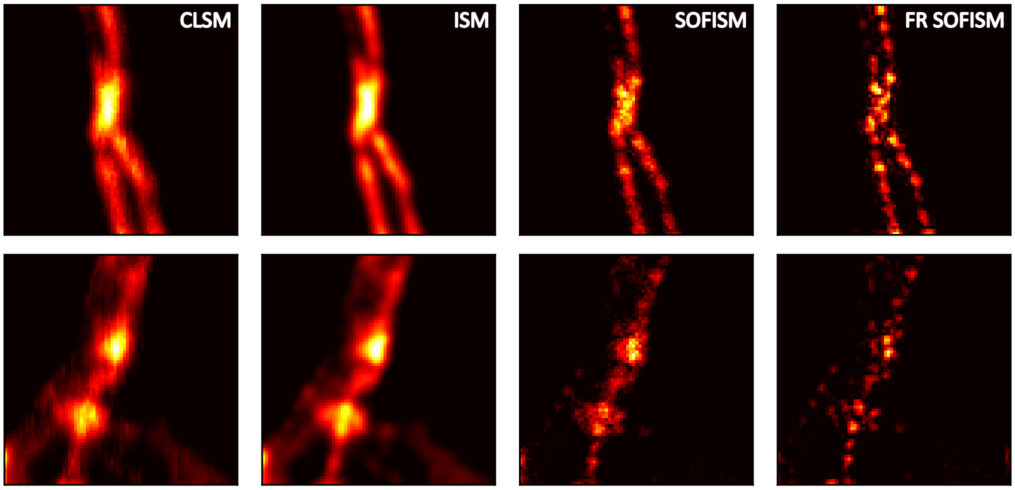

Fig. S7. Two examples of areas from a neuronal cell culture where actin is stained with Atto 643 dye. From left to tight, columns represent CLSM, ISM, SOFISM and FR-SOFISM images. In the first example (top), the main feature becomes sharper with every step without losing the integrity of the structure. In the bottom row, the appearance of the structure remains continuous in the SOFISM image. Following FR, some gaps in the linear filament can be observed. Scan pixel size is 50 nm.

REFERENCES

1. A. Sroda, A. Makowski, R. Tenne, *et al.*, "SOFISM: Super-resolution optical fluctuation image scanning microscopy," Optica **7**, 1308 (2020). ArXiv: 2002.00182 Publisher: OSA Place: Washington, D.C. ISBN: 978-1-943580-67-5.
2. T. Dertinger, R. Colyer, G. Iyer, *et al.*, "Fast, background-free, 3D super-resolution optical fluctuation imaging (SOFI)," Proc. Natl. Acad. Sci. **106**, 22287–22292 (2009).
